# Landscapes of the main components, metabolic and microbial signatures, and their correlations during stack “sweating” of *Eucommiae Cortex*

**DOI:** 10.3389/fmicb.2025.1550337

**Published:** 2025-02-28

**Authors:** Linfeng Wang, Mengxian Wu, Bingnan Gu, Erfeng Wang, Faliang Wu, Jiapeng Yang, Bing Guo, Xingke Li, Pengpai Zhang

**Affiliations:** ^1^School of Life Sciences, Institute of Microbial Engineering, Henan University, Kaifeng, China; ^2^Engineering Research Center for Applied Microbiology of Henan Province, Kaifeng, China; ^3^Henan Yangchen Pharmaceutical Company Limited, Sanmenxia, China; ^4^Henan Sanweiqi Food Limited Liability Company, Sanmenxia, China

**Keywords:** *Eucommiae Cortex*, mechanism of “sweating”, high-throughput sequencing, high performance liquid chromatography, untargeted metabolomics

## Abstract

**Introduction:**

“Sweating,” a key step in the processing and production of *Eucommiae Cortex* (EC), which plays a vital role in the formation of the medicinal quality of EC. However, the mechanism of the effect of this traditional treatment of herbs on the quality of herbs is still unclear.

**Methods:**

In this study, high performance liquid chromatography (HPLC), UPLC/MS-based untargeted metabolomics and high-throughput sequencing were applied to investigate the dynamic changes of the main active ingredients, differential metabolites and bacterial communities in the process of “sweating” in EC. The samples were prepared by the traditional stacking “sweating” method, and the samples were collected once a day for five consecutive days.

**Results:**

The results showed that the contents of the main active constituents, geniposidic acid (GPA), chlorogenic acid (CA), rutin (AU), pinoresinol diglucoside (PD) and total flavonoids (TFS), increased significantly after steaming, followed by a slight decrease. Furthermore, 807 metabolites were identified as crucial factors contributing to the metabolic alterations induced by the “sweating” process. Microbial diversity analysis showed considerable changes in microbiota characteristics, and the main functional microorganisms before and after “sweating” of EC were *Gluconobacter, unclassified_c_Gammaproteobacteria, Pseudomonas, Pantoea, Pedobacter, and Parecoccus*, which were involved in the five metabolic pathways of other secondary metabolites leading to significant changes in alkaloids, amino acid related compounds, flavonoids, phenylpropanoids and terpenoids.

**Discussion:**

The correlation network established between core bacterial communities, active ingredients, and metabolic pathways elucidates the microbial regulation of EC quality during sweating. These findings provide a scientific foundation for optimizing processing duration and advancing quality control strategies through targeted microbial community management.

## 1 Introduction

*Eucommia ulmoides* Oilv., a deciduous tree of the genus *Eucommia* in the family *Eucommiaceae*, is a famous medicinal and food plant with a long history in China (He et al., 2014). The bioactive chemical substances extracted from the dried bark, seeds, flowers and leaves of *Eucommia ulmoides* have high medicinal value, and *Eucommia ulmoides* has been used as health food and food additives in Japan, Korea, Russia, and the United Kingdom (Wang et al., 2011; He et al., 2014; Hosoo et al., 2017). *Eucommiae Cortex* (EC) contains lignans, cyclic enol ether terpenoids, flavonoids, phenylpropanoids, polyphenols, polysaccharides (PSs), and other pharmacologically active ingredients (Chen et al., 2010; Shahidi and Yeo, 2016), which have multiple pharmacological effects, such as lowering blood pressure, lowering blood lipids, lowering blood glucose, anti-tumour, anti-bacterial, anti-viral, protecting the kidneys and anti-osteoporosis (Li et al., 2013; Zhang R. et al., 2014). According to the Chinese Pharmacopoeia (edition 2020), the primary processing of EC should involve a method known as “sweating,” and it is preferable to sweat the inner skin to purple-brown colour, The total content of pinoresinol diglucoside (PD), geniposidic acid (GPA) and chlorogenic acid (CA) is used as the primary index to control the quality of EC (Zhang Q. et al., 2014; Li et al., 2015; Dong et al., 2022). More studies have found that the active ingredients and pharmacological effects of EC are stronger after “sweating” than those that have not undergone this process (Yan et al., 2018; Dai et al., 2022).

Studies have shown that “sweating” processing can redistribute the internal moisture of herbs, accelerate the drying rate; promote the biological and chemical transformation of medicinal components; and improve the appearance, texture, odour and medicinal properties of herbs, which in turn affects the pharmacological effects (Duan et al., 2013; Wang et al., 2016; Cao et al., 2020). Currently, some studies have demonstrated that a series of biochemical reactions, including oxidation, degradation, condensation, structural modification, methylation and glycosylation, occur within the herbs with the changes in temperature and humidity during the “sweating” process, which are directly related to the exogenous enzymes secreted by the microbiota (Guo et al., 2022). At the same time, it has also been found that the microflora of herbs is also in a state of dynamic change during the process of “sweating.” The dominant flora facilitates the biological and chemical transformation of both primary and secondary metabolites in herbs through metabolism process. This transformation produces new compounds or to participate in the regulation of enzymatic reactions within the tissues, thereby altering the chemical composition of the herbs and ultimately affecting the quality of the herbs (Ai et al., 2019; He et al., 2023; Wang et al., 2024) It has been found that the structure and diversity of microbial community of *Salvia miltiorrhiza* changed with the temperature and humidity during the process of “sweating,” and there was a certain correlation between the dominant microbial community and the improvement of the quality of *Salvia miltiorrhiza* herbs after the process of “sweating” (Wang et al., 2024). In addition, it was also found that the changes in ginsenoside content of red ginseng fermented with microbial strains were closely related to the specific microbial strains. The ginsenosides Re, Rg1 and Rb1 were reduced in fermented red ginseng, while the contents of ginsenosides Rh1, F2, Rg3 and compound Y (C.Y) were higher than those found in raw and ordinary red ginseng (Lee et al., 2015). Therefore, the effect of the “sweating” process on bioactive compounds in plants may be closely associated with related to microbial changes.

In recent years, EC has attracted extensive attention for its remarkable physiological activities. However, due to the varying origins and growing environments, unregulated processing, and ambiguous methods method of “sweating,” the quality of the herbs of EC on the market are uneven. These problems need to be solved through research. Meanwhile, previous studies on EC have primarily concentrated on the effects of different “sweating” methods on its active ingredients and efficacy (Yan et al., 2018), and there are very few studies on the study of its core microbiome (Dong et al., 2021). However, the mechanism of “sweating” of EC remains unclear. It is also unknown what role of microbial diversity and composition play in the overall “sweating” process. Therefore, it is crucial to understand the relationship between the core microorganisms and the main active ingredients in the “sweating” process of EC. Recent developments in metabolomics and microbiome have addressed this limitation. With the development of high-throughput sequencing technology, the composition and dynamics of the microbiota during the “sweating” process of various herbs have been elucidated (Wu et al., 2019; He et al., 2023). However, the active substances, as well as the unique characteristics of the microorganisms involved in the “sweating” process of EC, and the role of microbiota in the biotransformation of chemical components during this process, have not yet been fully constructed.

In this study, high performance liquid chromatography (HPLC) and untargeted metabolomics were used to determine the changes in the content of major active substances and secondary metabolites during the “sweating” process of EC. High-throughput sequencing was used to study the dynamic changes of bacterial communities during the “sweating” process of EC. At last, Spearman’s correlation coefficient was employed to analyze the effects of the bacterial community on the main active components and metabolites of EC. This study aimed to explore the relationship between the changes in active ingredients and metabolites and alterations in the microbial community during the process of “sweating” in EC. It is hoped to provide an atheoretical basis for exploring the mechanism of quality formation of EC herbs and standardizing the processing method of “sweating” in EC.

## 2 Materials and methods

### 2.1 Sample preparation and storage

Samples from *Eucommia ulmoides* plants with an age of more than are over 45 years old were collected in Jinzhai County, Anhui Province in May 2023. The bark at 1 m above the ground was peeled off and immediately taken back to the laboratory. The coarse bark of EC was then scraped off and washed with sterile water. The bark was cut into short sections measuring 5 cm by 3 cm using autoclaved scissors and then steamed in a cage drawer for 3 min. The inner surface of the bark was stacked on an ultra-clean workbench while it was still hot, covered with a black plastic bag, and “sweated” at room temperature. The inner surface of the skin of EC turns purplish-brown or brownish-brown as an indicator of the end of “sweating” of EC. “Sweating” was carried out for 5 days, during which samples were collected daily, resulting in a total of seven samples. These samples were labeled as follows: fresh sample (S0d), steamed sample (Sas), S1d, S2d, S3d, S4d, and S5d. The samples were promptly frozen at −80°C for subsequent use, with each sample being replicated three times to ensure accuracy.

### 2.2 Determination of the content of major chemical components in “sweating”

#### 2.2.1 Treatment of samples and extracts

The samples were placed in an oven at 50°C, dried to a constant weight, crushed, passed through a 40-mesh screen, and 0.5 g of each sample was accurately weighed. Ten milliliters of 50% methanol solution was added and thoroughly mixed, and then extracted by ultrasound (50°C, 400 W) for 60 min. After centrifugation at 8,000 r/min for 10 min, the supernatant was collected as the sample extract.

#### 2.2.2 Determination of polysaccharide content

PS content was determined by the phenol-sulfuric acid method using glucose as the standard. A volume of 0.1 mL of the extract was placed in an Eppendorf tube, followed by the addition of distilled water to replenish to 0.2 mL, and then 0.1 mL of 6% phenol solution was added. The samples were then shaken thoroughly, and 0.5 mL of concentrated H_2_SO_4_ was added, followed by additional shaking. The samples were sealed and placed at room temperature for 30 min, after which the absorbance value was measured at 490 nm (Wang et al., 2021).

#### 2.2.3 Determination of total phenol content

Total phenol (TP) content was determined by the Folin–Ciocalteu method using chlorogenic acid (Beijing Solarbio Science & Technology Co., Ltd.) as the standard (Singleton and Rossi, 1964). Five hundred microliters of the extract was mixed with 2.5 mL of Folin–Ciocalteu reagent (Beijing Solarbio Science & Technology Co., Ltd.) and the reaction was carried out for 3 min at room temperature. A 2 mL saturated Na_2_CO_3_ solution (20%, w/v) was added to the mixture, followed by incubation at room temperature in the dark for 90 min. The absorbance of the mixture was measured at 747 nm, and the total phenolic content was expressed as mg chlorogenic acid equivalents (mg CA)/g dry weight of the material.

#### 2.2.4 Determination of total flavonoids content

Rutin (Beijing Solarbio Science & Technology Co., Ltd.) as the standard, 1 mL of the extract was added into a 10 mL test tube. Three tenths of a milliliter of 5% NaNO_2_ solution was added, mixed thoroughly, and allowed to stand for 6 min. Next, 0.3 mL of 10% aluminum nitrate solution was added, shaken well, and left an additional 6 min. Then, 4 mL of 1 mol/L NaOH solution was added, and then diluted to 10 mL with 30% ethanol solution, followed by thorough shaking. Samples were allowed to stand for 15 min and the absorbance was measured at 510 nm (Shao and Li, 2013).

### 2.3 Quantitative analysis of chlorogenic acid, genipinic acid, pinosyl diglucoside, and rutin

The standards chlorogenic acid (CA) (≥98%), genipinic acid (GPA) (≥98%), pinosyl diglucoside (PD) (≥98%), and rutin (AU) (≥98%) were purchased from Beijing Solarbio Science and Technology Co., Ltd. (Beijing, China). Analyses were performed with slight modifications to the previously described chromatographic conditions (Ding et al., 2014). Sample extracts prepared in 2.3.1 were collected and filtered through a 0.22 μm nylon filter (Zinteng, China), and then 1 mL of the supernatant was analysed by HPLC. The separation was performed on a Welch Boltimate C18 column (100 mm × 4.6 mm, 2.7 μM, Yuexu Technology Shanghai Co.) using water 1525 (Waters, United States) with gradient elution. Chromatographic conditions: injection volume: 10 μL; UV detection at 238 nm; mobile phase A, acetonitrile; mobile phase B: water plus 0.1% (v/v) acetic acid; column temperature: 25°C; flow rate: 1 mL/min; gradient elution, 5–8% B, 0–12 min; 8–15% B, 12–20 min; 15–20% B, 20–30 min; 20–30% B, 30–40 min; 30–5% B, 40–45 min; 5–5% B, 45–47 min. The identification of compounds was carried out by comparing their retention times with those of reference standards. Quantification was performed using external calibration curves established from the peak areas of the corresponding reference standards analyzed under identical chromatographic conditions.

### 2.4 Untargeted metabolomics

An ultra performance liquid chromatography-tandem mass spectrometry (UPLC-MS/MS)-based metabolomics method was used to extract and analyze the metabolites in EC samples (Majorbio bio-pharma Technology Co., Ltd.). A 100 mg sample was accurately weighed, and 400 μL of cold methanol solution (methanol: water = 1:4) was added. The sample was broken by a high-throughput tissue crusher at low temperature, a vortex tissue crusher, vortex-mixed, and ultrasonicated for 30 min on ice. The samples were subsequently stored at −20°C for 30 min, and then centrifuged at 1300 g for 15 min at 4°C. Finally, the supernatant was taken and filtered through a 0.22 μm filter for UPLC-MS/MS analysis.

#### 2.4.1 LC-MS assay conditions

UPLC-MS/MS analysis was performed on an ultra-high performance liquid chromatography tandem time-of-flight mass spectrometry UPLC-TripleTOF system from AB SCIEX, with a BEH C18 column (100 mm × 2.1 mm i.d., 1.7 μm; Waters, Miford, United States); the mobile phase A was water (containing 0.1% formic acid) and the mobile phase B was acetonitrile/isopropanol (1/1) (containing 0.1% formic acid); the gradient elution procedure was 0–3 min: 0–20% B, 3–9 min: 20–60% B, 9–11 min: 60–100% B, 100% B was maintained for 2.5 min, 13.5–13.6 min: 100 to 0% B, and 0% B was maintained for 2.4 min. The flow rate was 0.40 mL/min, the injection volume was 20 μL, and the column temperature was set at 40°C.

Sample mass spectrometry signals were acquired using both positive and negative ion scanning modes, respectively, with electrospray capillary voltage, injection voltage and collision voltage of were set at 1.0 kV, 40 V, and 6 eV, respectively, ionization and desolventization temperatures of 120°C and 500°C, respectively, while the and a carrier gas flow rate of 900 L/h, with a mass spectrometry scanning range of 50 to 1,000 *m*/*z* and a resolution of 30,000.

#### 2.4.2 Untargeted metabolomics data analysis

For untargeted metabolomics data, a number of columns of preprocessing of the raw data are required before conducting statistical analysis. Raw data were imported into the metabolomics processing software Progenesis QI (Waters Corporation, Milford, United States) for baseline filtering, peak identification, integration, retention time correction, peak alignment, and then data preprocessing to obtain the final data matrix for subsequent analysis. The original data were searched and identified by Progenesis QI software, and the MS and MS/MS mass spectrum information was matched with the metabolic database. Unsupervised principal component analysis (PCA) was used to observe the population distribution of each sample and the degree of dispersion between groups. Supervised (orthogonal) partial least squares analysis (PLS-DA) was then used to distinguish the overall differences in metabolic profiles between groups and find differential metabolites between groups.

### 2.5 High-throughput sequencing

#### 2.5.1 DNA extraction

The microbial communities of the samples were examined according to Wu et al. (2019) with slight modifications. Microbial DNA was extracted from *Eucommia ulmoides* bark samples using the E.Z.N.A.^®^ Soil DNA Kit (Omega Biotek, Inc., Doravilla, GA, United States) according to the manufacturer’s protocol. The quality of DNA extracts from EC samples was checked on 1% agarose gels. Subsequently, DNA concentration and purity were determined using a NanoDrop 2000 UV–visible spectrophotometer (Thermo Fisher Scientific, Wilmington, United States).

#### 2.5.2 16S rRNA amplification and sequencing

An ABI GeneAmp^®^ 9700 PCR thermocycler (ABI, CA, United States) was used. The V5–V7 region of the 16S rRNA gene was amplified with 779F (5′-AACMGGATTAGATACCCKG-3′) and 1193R (5′-ACGTCATCCCCACCTTCC-3′) primers (Qiu et al., 2020). PCR amplification of the 16S rRNA gene: Initial denaturation step at 95°C for 3 min, followed by cycles of denaturation at 95°C for 30 s, annealing at 55°C for 30 s, extension at 72°C for 45 s, a single extension at 72°C for 10 min, and the reaction was held at 10°C until completion. The cycle number of 799F-1392R for one round of primers was 27, and the cycle number of 799F-1193R for two rounds was 13. The PCR reaction system was as follows: 4 μL of 5 × FastPfu Buffer, 2 μL of 2.5 mol/L dNTPs, 0.8 μL each of forward and reverse primers (5 μmol/L), 0.4 μL of (2.5 U/μL) TransStartFastPfu Polymerase, 0.2 μL BSA (0.8 μg/μL), 10 ng Template DNA, with ddH2O supplemented to 20 μL.

#### 2.5.3 Illumina sequencing and bioinformatics processing

According to Majorbio Bio-pharma Technology Co., Ltd. the standard protocol pooled equal amounts of purified PCR products. Paired-end sequencing was performed by using an Illumina Miseq PE300 platform (Illumina, San Diego, United States). The primer sequences were removed. Fastp version 0.20.0 (Chen, 2023) was used for unmultiplex and quality screening of raw 16S rRNA gene sequencing fragments, and FLASH version 1.2.7 was used for merging (Magoč and Salzberg, 2011). Operational taxonomic units (OTUs) of non-repetitive sequences (excluding individual sequences) were clustered based on 97% similarity using UPARSE software (https://drive5.com/uparse/, version 7.1) (Stackebrandt and Goebel, 1994). Chimeras were identified and removed during the clustering process. Each OTU representative sequence was annotated and analyzed for species classification against the Silva 16S rRNA database using the RDP classifier (https://rdp.cme.msu.edu/, version 2.2) (Wang et al., 2007) with a confidence threshold of 0.7. α diversity indices including Shannon, Simpson, Ace, Chao1, and Good’s coverage were assessed using Mothur software (version 1.30.2). β diversity analysis was performed using QIIME software (version 1.9.1) and visualized by principal coordinate analysis (PCoA). Bacterial taxa with significant differences in abundance from phylum to genus level between groups were identified using Linear discriminant analysis Effect Size analysis (LDA >2, *p* < 0.05), and bacterial genera with the highest ranked abundance levels were selected for further correlation analysis.

### 2.6 Correlation analysis

The microbial metabolism, active ingredients and metabolites detected during the “sweating” process of EC were analyzed for correlation. Spearman’s algorithm was employed to create heat maps of 10 core microbial genera with seven active ingredients and various metabolites. Spearman’s correlation values ranged between −1 and +1. Red colour indicates a positive correlation of *r* > 0 and blue colour indicates a negative correlation of *r* < 0. A *p*-value of less than 0.05 indicates a significant correlation.

### 2.7 Statistical analysis

Data were expressed as the mean ± standard deviation (*n* = 3) and plotted using Origin2021 (OriginPro Lab Corp., Northampton, United States). Significance analyses were performed using SPSS 27.0 (IBM SPSS, United States), with *p* < 0.05 indicating a statistically significant difference.

## 3 Results

### 3.1 Changes of major components during sweating of EC

**Figure 1:**
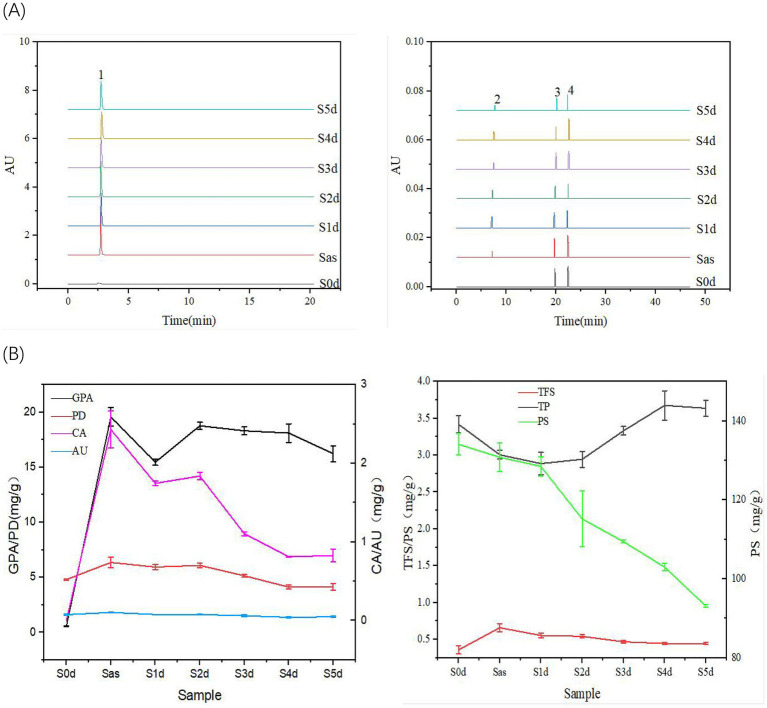
Superimposed fingerprint of *Eucommia ulmoides* in over the sweating timecourse **(A)**, in which 1 is GPA, 2 is CA, 3 is PD, and 4 is AU. Changes of GPA, PD, CA, AU, PS, TFS, and TP content in *Eucommia ulmoides* during the sweating timecourse **(B)**.

The dynamic changes in the active ingredients of EC samples S0d, Sas, S1d, S2d, S3d, S4d and S5d were compared and analyzed. The results are shown in Figure 1 and Table 1. The content of PS decreased from S0d to S5d. The content of PS in S0d samples was 134.100 ± 2.760 mg/g, while in S5d samples, it decreased to 93.121 ± 0.345 mg/g. This may be attributed to the fact that microorganisms utilize sugars for metabolic activities during the “sweating” process, resulting in the production of substances such as lactic acid and alcohol (Zhang Q. et al., 2014). The TP content of the samples decreased after steaming, but showed a tendency of initially increasing and then decreasing during the “sweating” proceeded, and the TP content was 2.886 ± 0.150 mg/g on the first day of sweating (S1d), and reached a maximum value of 3.675 ± 0.198 mg/g on the fourth day of sweating (S4d). On the contrary, the total flavonoids (TFS) content increased rapidly after steaming, and then decreased gradually with the “sweating,” and the TFS content was 0.555 ± 0.032 mg/g on the first day of “sweating” (S1d), and decreased to 0.445 ± 0.016 mg/g on the fifth day of “sweating” (S5d). The total phenolic content and total flavonoid content were higher than those of the fresh samples at the end of the sweating (S0d), although there were some changes during the process of “sweating.” This phenomenon occurs because the active components of EC are transformed into other substances due to the action of microorganisms during “sweating.”

**Table 1:** Identification of active compounds (*n* = 3).

				Compounds (mg/g)			
Sweating time	GPA	CA	AU	PD	PS	TP	TFS
S0d	0.550 ± 0.022^d^	0.000 ± 0.000^e^	0.075 ± 0.007^bc^	4.803 ± 0.068^b^	134.100 ± 2.760^a^	3.420 ± 0.115^bc^	0.358 ± 0.056^d^
Sas	19.577 ± 0.838^a^	2.430 ± 0.236^a^	0.104 ± 0.001^a^	6.360 ± 0.465^a^	130.808 ± 3.624^a^	3.006 ± 0.058^d^	0.663 ± 0.056^a^
S1d	15.467 ± 0.285^c^	1.746 ± 0.029^b^	0.077 ± 0.001^b^	5.941 ± 0.242^a^	128.489 ± 2.416^a^	2.886 ± 0.150^d^	0.555 ± 0.032^b^
S2d	18.772 ± 0.314^ab^	1.838 ± 0.045^b^	0.077 ± 0.002^b^	6.090 ± 0.199^a^	115.196 ± 7.071^b^	2.945 ± 0.109^d^	0.543 ± 0.024^b^
S3d	18.319 ± 0.362^b^	1.105 ± 0.024^c^	0.062 ± 0.016^cd^	5.148 ± 0.122^b^	109.453 ± 0.361^bc^	3.330 ± 0.058^c^	0.469 ± 0.014^c^
S4d	18.113 ± 0.838^b^	0.812 ± 0.007^d^	0.042 ± 0.006^e^	4.117 ± 0.173^c^	103.000 ± 0.863^c^	3.675 ± 0.198^a^	0.445 ± 0.016^c^
S5d	16.240 ± 0.729^c^	0.827 ± 0.081^d^	0.049 ± 0.006^de^	4.155 ± 0.308^c^	93.121 ± 0.345^d^	3.636 ± 0.110^ab^	0.445 ± 0.014^c^

Different lowercase letters in the superscripts of the same line (a–e) indicate significant differences in the active ingredients in the one-way ANOVA. (*p* < 0.05).

In addition, the content of PD, which serves a standard for evaluating the quality of EC, undergoes significant changes during “sweating.” The content of PD increased rapidly from 4.803 ± 0.068 mg/g (S0d) to 6.360 ± 0.465 mg/g (Sas) after steaming, then decreased from Sas to S1d to 5.941 ± 0.242 mg/g, and then increased to 6.090 ± 0.199 mg/g (S2d), From S2d to S5d, the content slowly decreased to 4.155 ± 0.308 mg/g (S5d). The changes of GPA, CA, AU and PD contents were basically the same. The content of GPA increased rapidly from 0.550 ± 0.022 mg/g (S0d) to 19.577 ± 0.838 mg/g (Sas) after evaporation, and then decreased from Sas to S1d to 19.577 ± 0.838 mg/g, then to 18.772 ± 0.314 mg/g (S2d), and then showed a slow decreasing trend from S2d to S5d, and finally decreased to 16.240 ± 0.729 mg/g. The presence of CA was not detected in the fresh samples, and after steaming, the presence of CA was found to reach 2.430 ± 0.236 mg/g (Sas), and then decreased from Sas to 1.746 ± 0.029 mg/g (S1d), then increased to 1.838 ± 0.045 mg/g (S2d), and then showed a decreasing trend from S2d to S5d, and finally decreased to 0.827 ± 0.081 mg/g (S5d). The content of AU increased rapidly from 0.075 ± 0.007 mg/g (S0d) to 0.104 ± 0.001 mg/g (Sas) after evaporation, and then gradually decreased to 0.049 ± 0.006 mg/g from Sas to S5d. All four active ingredients were affected by steaming, which might be related to the water overflow of the EC samples during the steaming process. At the same time, the cell wall of EC was destroyed during the high-temperature steaming process, resulting in the dissolution of a significant number of active ingredients. In the process of “sweating,” the endogenous microorganisms would play an important role in promoting the changes of enzyme activities within the samples, which subsequently leads to the transformation of the active ingredients.

### 3.2 Changes of metabolites during sweating in EC

Due to the complexity of the chemical composition during “sweating” in EC, secondary metabolite analyses were insufficient. In order to further elucidate the metabolite profiles during “sweating,” an untargeted metabolomics analysis based on UPLC-MS/MS was conducted.

**Figure 2:**
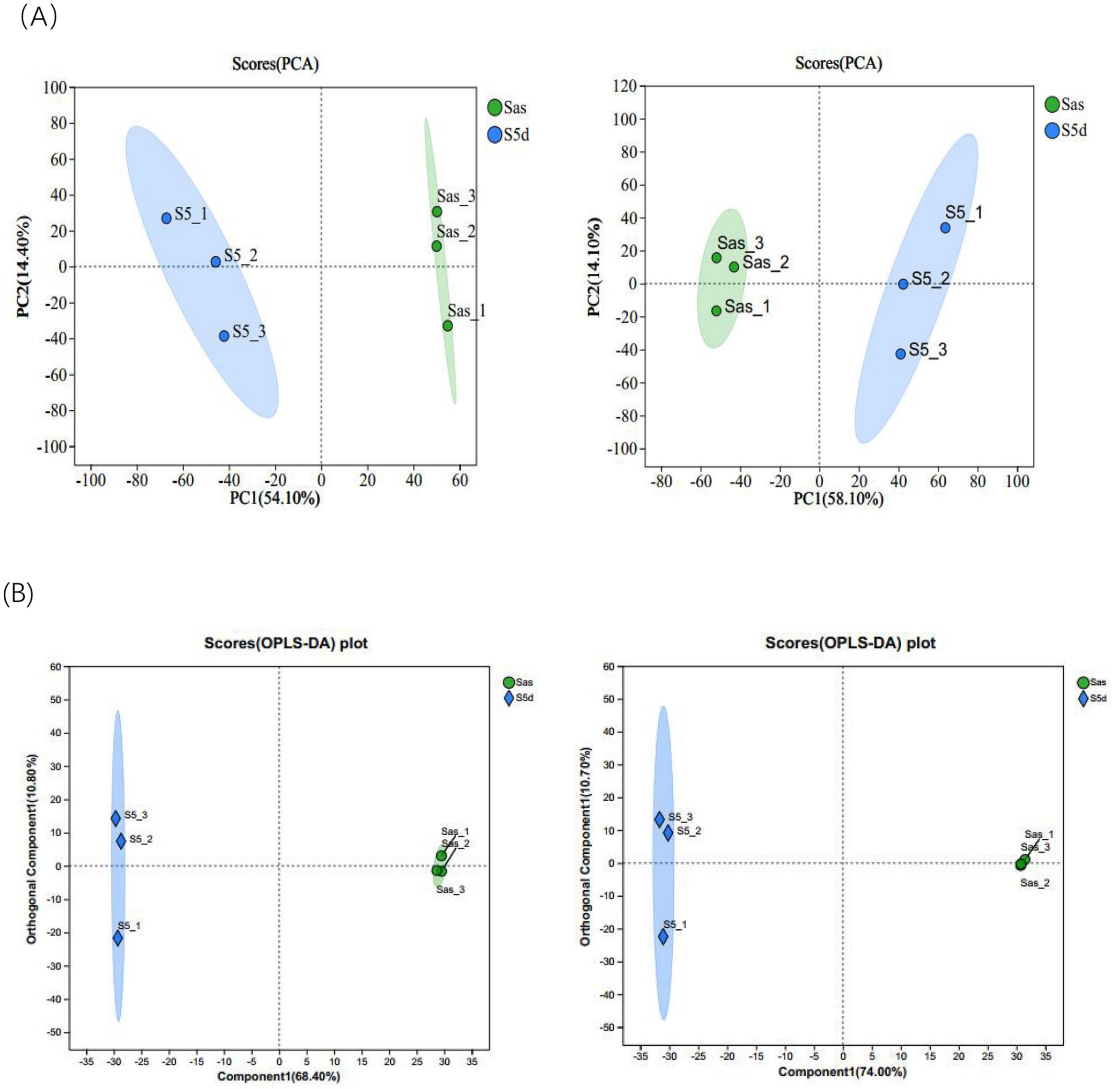
**(A)** Plots of PCA scores in positive and negative ion modes. **(B)** Plot of OPLS-DA scores in positive and negative ion modes.

Six samples of EC before and after “sweating” were analyzed by LC-MS/MS. The positive and negative ion modes, respectively, retained 356 and 434 components. Subsequently, 172 positive ion component names and 218 negative ion component names were successfully identified in the KEGG compound database. Principal component analysis was conducted on the samples before and after “sweating,” as shown in Figure 2A, there was a large distance between the samples after steaming (Sas) and the samples after “sweating” (S5d), which reflected that there were large differences in metabolic components between the two groups. This result provided basic support for the subsequent screening of differential metabolites. The orthogonal partial least squares-discriminant analysis (OPLS-DA) was used to predict the components of the two groups. Figure 2B show that there are significant differences between the two groups. Furthermore, all samples are within the 95% confidence interval, indicating that the data obtained from this experiment possess both authenticity and reliability.

**Figure 3:**
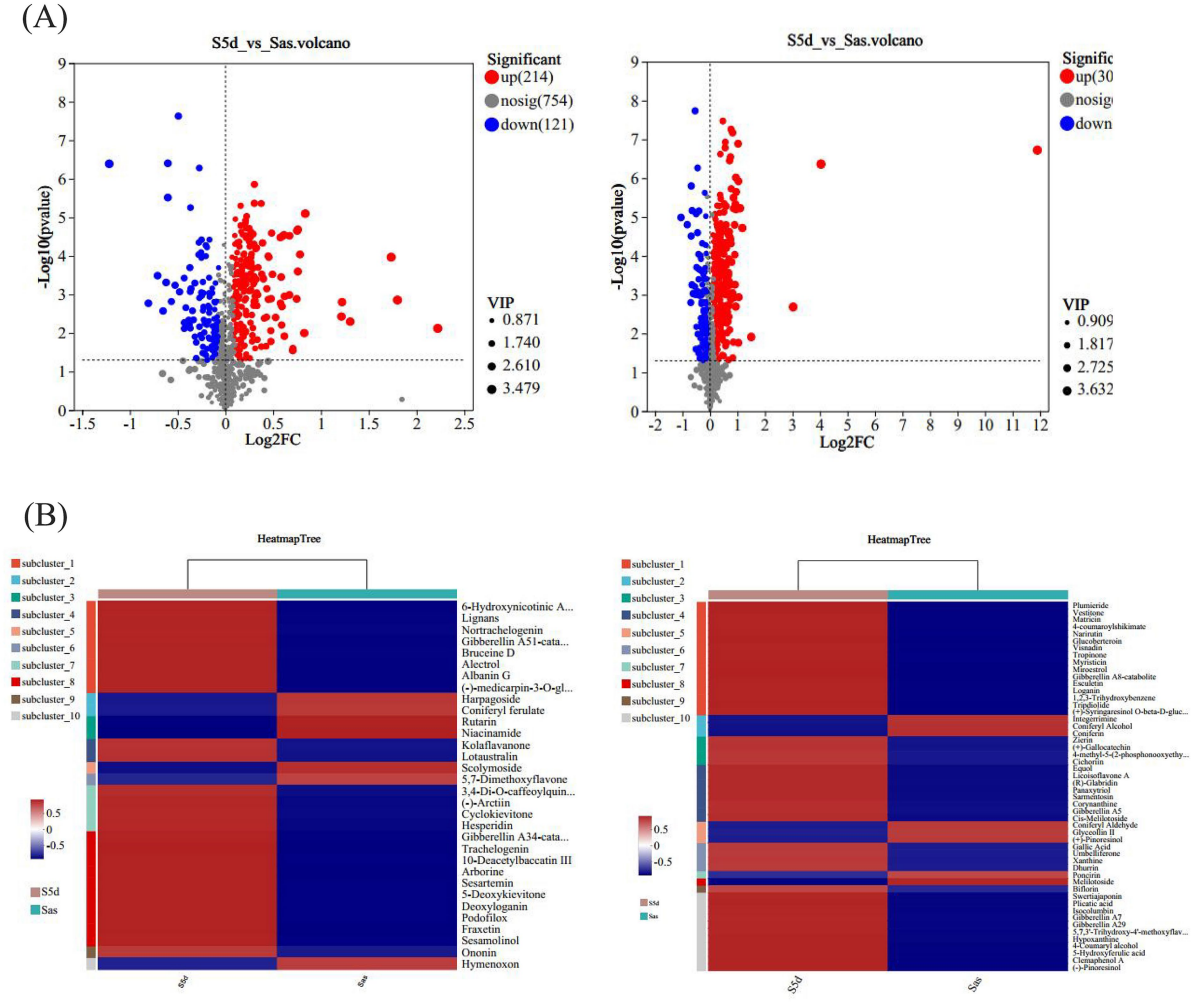
**(A)** Volcano plots of differential components in positive and negative ion modes. **(B)** Heatmap of differential components clustering in positive and negative ion modes.

In order to screen out differential metabolic fractions, a combination of both *p*-values and VIP-values from the OPLS-DA model was utilized, as illustrated in Figure 3A. Each point represents a distinct metabolic component, the red points represent the significantly up-regulated component, the blue points represent the significantly down-regulated component, and the gray points represent the components with no significant differences. The results showed that 214 up-regulated metabolites and 121 down-regulated metabolites were detected in the positive ion mode, while 307 up-regulated metabolites and 123 down-regulated metabolites were detected in the negative ion mode. The screened compounds were compared with the KEGG compound database to identify compounds with definite names. As shown in Tables 2, 3, there were 27 differential compounds in the positive ion mode, of which 21 differential components were significantly up-regulated and six differential components were significantly down-regulated. A total of 46 differential components were identified in the negative ion mode, of which 38 were significantly up-regulated and eight were significantly down-regulated. The differential components were mainly alkaloids, amino acid related compounds, flavonoids, phenylpropanoids, polyketones and terpenoids.

**Table 2:** Differential components in the positive ion mode.

Metabolite	Regulate	Formula	Exact. mass	Categories
6-Hydroxynicotinic Acid	Up	C_6_H_5_NO_3_	140.0342	Alkaloids
Arborine	Up	C_16_H_14_N_2_O	295.0811	Alkaloids
Lotaustralin	Up	C_11_H_19_NO_6_	226.1073	Amino acid related compounds
Kolaflavanone	Up	C_31_H_24_O_12_	589.1285	Flavonoids
Albanin G	Up	C_45_H_44_O_11_	783.2839	Flavonoids
Hesperidin	Up	C_28_H_34_O_15_	575.1732	Flavonoids
Ononin	Up	C_22_H_22_O_9_	463.1572	Flavonoids
Cyclokievitone	Up	C_20_H_18_O_6_	387.1434	Flavonoids
5-Deoxykievitone	Up	C_20_H_20_O_5_	341.1381	Flavonoids
Fraxetin	Up	C_10_H_8_O_5_	191.0338	Phenylpropanoids
Trachelogenin	Up	C_21_H_24_O_7_	371.1485	Phenylpropanoids
(−)-Arctiin	Up	C_27_H_34_O_11_	552.2439	Phenylpropanoids
Lignans	Up	C_22_H_22_O_8_	415.1384	Phenylpropanoids
Sesartemin	Up	C_23_H_26_O_8_	431.1683	Phenylpropanoids
Podofilox	Up	C_22_H_22_O_8_	447.1624	Phenylpropanoids
Sesamolinol	Up	C_20_H_20_O_7_	373.1271	Phenylpropanoids
3,4-Di-O-caffeoylquinic acid	Up	C_25_H_24_O_12_	499.1234	Phenylpropanoids
10-Deacetylbaccatin III	Up	C_29_H_36_O_10_	586.2646	Terpenoids
Deoxyloganin	Up	C_17_H_26_O_9_	413.1203	Terpenoids
Alectrol	Up	C_19_H_22_O_6_	311.1274	Terpenoids
Bruceine D	Up	C_20_H_26_O_9_	433.147	Terpenoids
Niacinamide	Down	C_6_H_6_N_2_O	123.0555	Alkaloids
5,7-Dimethoxyflavone	Down	C_17_H_14_O_4_	315.1224	Flavonoids
Scolymoside	Down	C_27_H_30_O_15_	627.1896	Flavonoids
Rutarin	Down	C_20_H_24_O_10_	425.1415	Phenylpropanoids
Coniferyl ferulate	Down	C_20_H_20_O_6_	357.1329	Phenylpropanoids
Harpagoside	Down	C_24_H_30_O_11_	512.2125	Terpenoids

**Table 3:** Differential components in the negative ion mode.

Metabolite	Regulate	Formula	Exact. Mass	Categories
Corynanthine	Up	C_21_H_26_N_2_O_3_	389.1608	Alkaloids
4-methyl-5-(2-phosphonooxyethyl)thiazole	Up	C_6_H_10_NO_4_PS	259.9549	Alkaloids
Xanthine	Up	C_5_H_4_N_4_O_2_	151.0254	Alkaloids
Hypoxanthine	Up	C_5_H_4_N_4_O	135.0304	Alkaloids
Zierin	Up	C_14_H_17_NO_7_	667.1952	Amino acid related compounds
Sarmentosin	Up	C_11_H_17_NO_7_	595.2039	Amino acid related compounds
Dhurrin	Up	C_14_H_17_NO_7_	292.0829	Amino acid related compounds
Glucoberteroin	Up	C_13_H_24_NO_9_S_3_^−^	469.027	Amino acid related compounds
Panaxytriol	Up	C_17_H_26_O_3_	323.1864	Fatty acids related compounds
(+)-Gallocatechin	Up	C_15_H_14_O_7_	351.0723	Flavonoids
Swertiajaponin	Up	C_22_H_22_O_11_	507.1146	Flavonoids
Narirutin	Up	C_27_H_32_O_14_	561.1619	Flavonoids
5,7,3′-Trihydroxy-4′-methoxyflavanone	Up	C_16_H_14_O_6_	603.1553	Flavonoids
Licoisoflavone A	Up	C_20_H_18_O_6_	353.1031	Flavonoids
(R)-Glabridin	Up	C_20_H_20_O_4_	369.1344	Flavonoids
Vestitone	Up	C_16_H_14_O_5_	331.0824	Flavonoids
Gallic Acid	Up	C_7_H_6_O_5_	169.0136	Others
1,2,3-Trihydroxybenzene	Up	C_6_H_6_O_3_	125.0236	Others
Visnadin	Up	C_21_H_24_O_7_	387.145	Phenylpropanoids
Esculetin	Up	C_9_H_6_O_4_	177.0187	Phenylpropanoids
Umbelliferone	Up	C_9_H_6_O_3_	161.0237	Phenylpropanoids
Cichoriin	Up	C_15_H_16_O_9_	385.0778	Phenylpropanoids
Plicatic acid	Up	C_20_H_22_O_10_	403.1036	Phenylpropanoids
(−)-Pinoresinol	Up	C_26_H_32_O_11_	501.177	Phenylpropanoids
4-coumaroylshikimate	Up	C_16_H_16_O_7_	301.0717	Phenylpropanoids
Myristicin	Up	C_11_H_12_O_3_	191.0708	Phenylpropanoids
4-Coumaryl alcohol	Up	C_9_H_10_O_2_	345.1344	Phenylpropanoids
5-Hydroxyferulic acid	Up	C_10_H_10_O_5_	209.0452	Phenylpropanoids
Biflorin	Up	C_16_H_18_O_9_	707.1838	Polyketides
Isocolumbin	Up	C_20_H_22_O_6_	403.14	Terpenoids
Gibberellin A5	Up	C_19_H_22_O_5_	329.1395	Terpenoids
Gibberellin A7	Up	C_19_H_22_O_5_	375.145	Terpenoids
Gibberellin A29	Up	C_19_H_24_O_6_	393.1557	Terpenoids
Tripdiolide	Up	C_20_H_24_O_7_	421.1506	Terpenoids
Plumieride	Up	C_21_H_26_O_12_	515.1407	Terpenoids
Loganin	Up	C_17_H_26_O_10_	435.1509	Terpenoids
Matricin	Up	C_17_H_22_O_5_	327.1239	Terpenoids
Miroestrol	Up	C_20_H_22_O_6_	403.1398	Terpenoids
Integerrimine	Down	C_18_H_25_NO_5_	372.1215	Alkaloids
Poncirin	Down	C_28_H_34_O_14_	593.1883	Flavonoids
Glyceollin II	Down	C_20_H_18_O_5_	337.1083	Flavonoids
(+)-Pinoresinol	Down	C_20_H_22_O_6_	357.1343	Phenylpropanoids
Coniferyl aldehyde	Down	C_10_H_10_O_3_	177.0551	Phenylpropanoids
Melilotoside	Down	C_15_H_18_O_8_	325.0929	Phenylpropanoids
Coniferyl alcohol	Down	C_10_H_12_O_3_	179.0708	Phenylpropanoids
Coniferin	Down	C_16_H_22_O_8_	387.1297	Phenylpropanoids

A total of seven were screened out of all differential alkaloid fractions. 6-Hydroxynicotinic acid, one of the alkaloids, is an organic compound with a wide range of applications. It exhibits numerous many biological activities in the pharmaceutical field, including anti-tumor, anti-oxidation, lipid-lowering, and anti-inflammatory, and can prevent and treat a variety of diseases (Shang et al., 2021). Xanthine and hypoxanthine are the precursor molecules of nucleotides and nucleic acids, which play important roles in gene synthesis and delivery. Five amino acid-related compounds were screened, mainly including lotaustralin, zirin, sarmentosin, dhurrin, and glucoberteroin, all of which were significantly up-regulated. Lotaustralin exhibits a diverse array of pharmacological activities, including antioxidant, antibacterial, and anti-inflammatory properties, as well as the ability to promote wound healing. Due to its broad applicability, it is frequently utilized as a nutraceutical ingredient. Its benefits include the enhancement of blood circulation, the facilitation of collagen synthesis, and the stimulation of skin cell proliferation, all of which positively contribute positively to skin health and beauty. Fan et al. (2022) studied the effects of salmentosin on liver tissue morphology and PERK/eIF2α communication pathways in rats with biliary obstruction, and found that salmentosin improved the pathological morphology and oxidative stress levels of the liver tissue and reduced hepatocyte apoptosis. There were 17 flavonoid fractions that differed before and after “sweating,” with 13 flavonoids being up-regulated and four down-regulated. These flavonoids included hesperidin, ononine, (+)-gallic acid and naringin. Hesperidin may exert anti-osteoporotic effects by decreasing inflammatory cytokines and oxidative stress response (Yi et al., 2024). Ononin has antioxidant, anti-inflammatory, antitumor, immunomodulatory and antithrombotic effects (Zhang et al., 2024). The (+)-gallic acid may exert anticancer effects by inhibiting tumor cell growth and migration, regulating tumor cell apoptosis, and promoting tumor cell autophagy (Cui et al., 2015). Fraxetin and lignin in phenylpropanoids; among the terpenoids, brucine D and loganin have pharmacological activities such as anti-tumor, antihypertensive, anti-inflammatory, antioxidant and anti-cancer (Cui et al., 2015; Yi et al., 2024; Zhang et al., 2024). These metabolites are significantly up-regulated following the “sweating,” indicating that this process will lead to significant changes in the active components of EC and has a significant impact on the formation of the quality of the medicinal materials. At the same time, the selected differential components were analyzed by cluster heat map (Figure 3B), which also showed that there were significant differences between the two groups of samples, and most of the differential metabolites were significantly up-regulated after “sweating.”

**Figure 4:**
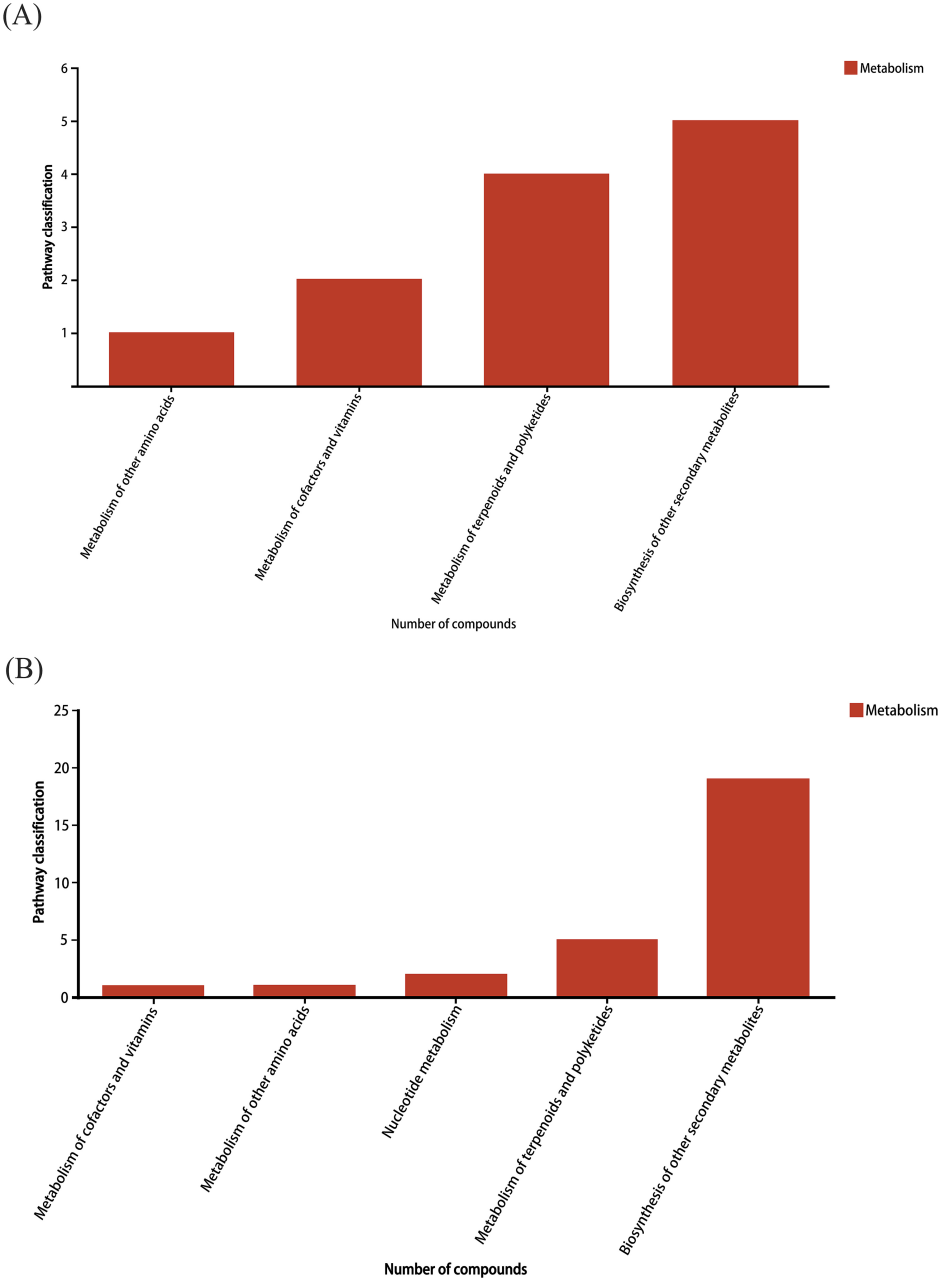
Bar plots of pathway types of differential components in **(A)** positive and **(B)** negative ion modes.

The differential components were compared with the KEGG pathway database to obtain the KEGG pathway statistical map, as shown in Figure 4. In this figure, the *x*-axis represents the secondary classification of the KEGG metabolic pathway, while the *y*-axis indicates the number of metabolites annotated within each pathway. Figure 4A shows that the metabolic classification of differential components in the positive ion mode is mainly the biosynthesis of other secondary metabolites, the metabolism of terpenoids and polyketones, the metabolism of cofactors and vitamins, and the metabolism of other amino acids. Figure 4B shows that the metabolic classification of differential components in the negative ion mode is mainly the biosynthesis of other secondary metabolites, the metabolism of terpenoids and polyketones, the metabolism of nucleotide, the metabolism of other amino acids, and the metabolism of cofactors and vitamins.

### 3.3 Changes of microbial communities during sweating in EC

#### 3.3.1 Microbial diversity and richness

**Figure 5:**
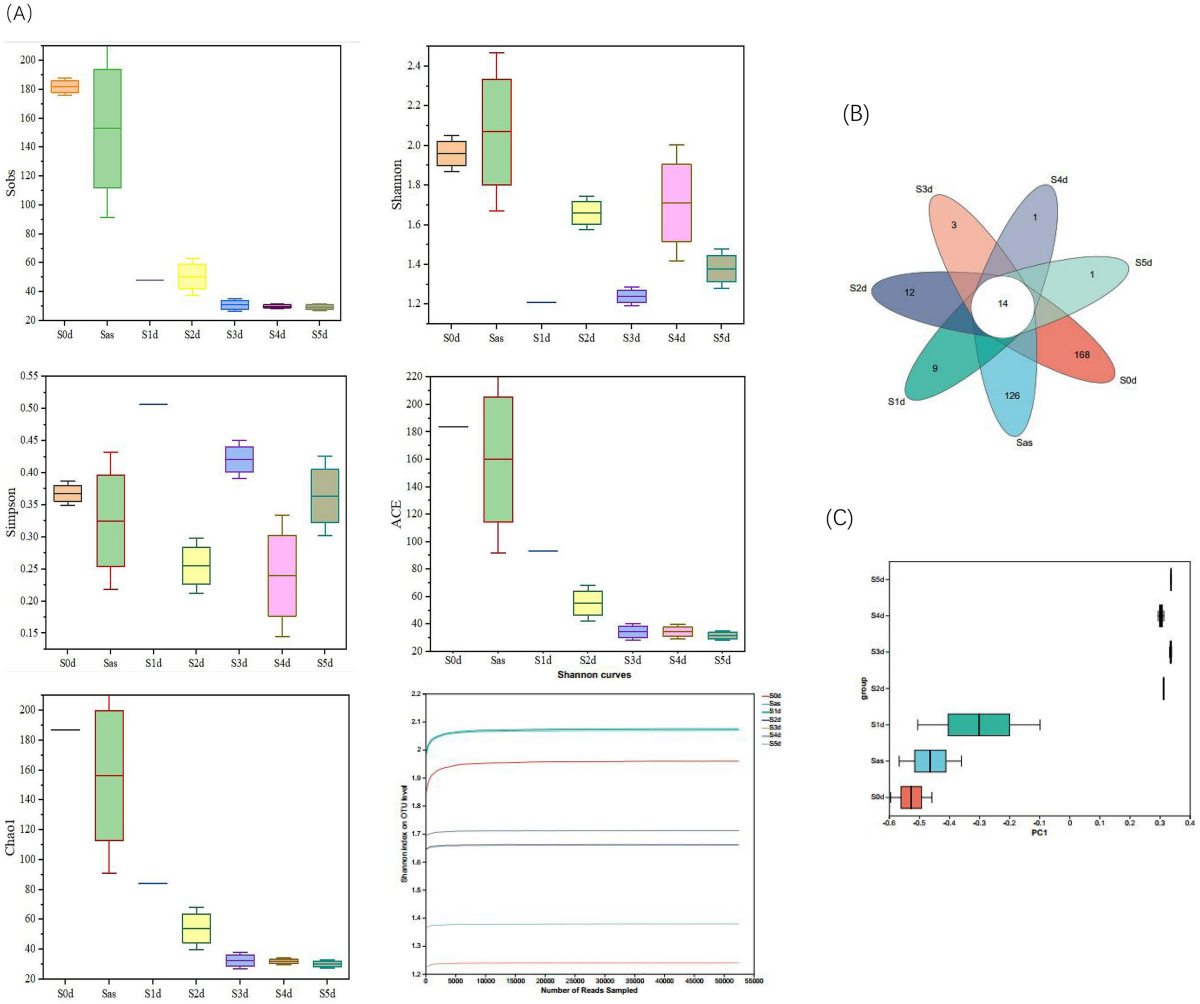
Microbial diversity during the *Eucommia ulmoides* sweating process. **(A)** α diversity, **(B)** Venn diagram, and **(C)** PCoA analysis of β diversity based on OTU abundance of microbial communities. S0d, Sas, S1d, S2d, S3d, S4d, and S5d represent the seven sampling points of the whole sweating timecourse, respectively.

An Illumina Miseq^™^ high-throughput sequencing platform was used to sequence the amplified products of bacterial 16S rRNA gene (s). A total of 1,089,978 original sequences were obtained from seven sampling times. The original sequences were preprocessed and spliced, and the length distribution ranging from 204 bp to 430 bp. After processing, the number of sequences and the average length of sequences in each group decreased, and 1,070,682 sequences were obtained, with an average sequence length of about 376 bp. All samples were undergone OTU cluster analysis at the 97% similarity level. A total of 602 OTUs were obtained from the seven sampling times, with the number of each group ranging from 27 to 306, with an average of 93 OTUs per sample. The sequencing coverage of the samples at each stage was >0.99, indicating that nearly all sequence types were detected in these samples, and the sequencing results could comprehensively characterize the bacterial communities across all samples. The α diversity index was used to evaluate the diversity and richness of the microbiota. Figure 5 illustrates the changes in bacterial diversity before, during “sweating,” and afterwards in EC. During the “sweating” process, the overall Shannon index showed a decreasing trend, while the Simpson index showed an opposite trend (Figure 5A). This indicates that during the “sweating” process from days 1 to 5, the microbioal diversity of EC was the highest in the pre-sweating period, and then decreased. The Sobs, ACE, and Chao 1 indices decreased rapidly on day 1 with the duration of sweating, then gradually decreased and leveled off. As expected, in S0d samples, the bacterial community was most abundant. Bacterial diversity and richness decreased after “sweating” during S1d to S5d. Lv et al. (2020) suggested that an OTU represents a microbial species and that changes in the bacterial community can be demonstrated by changes in OTU abundance. A Venn diagram (Figure 5B) showed that there were 14 OTUs in common across the 7 “sweating” stages and the unique OTUs decreased from 9 to 1 from S1d to S5d, indicating that these 14 OTUs were dominant during Eucommia “sweating.” To better characterize the changes and similarities in community composition, the β diversity index was calculated and evaluated using principal coordinate analysis (PCoA) based on OTU levels (Figure 5C). The PCoA results showed that the bacterial community composition was dramatically different at the timepoints from 2 days after steaming. This result suggested that critical effects on the bacterial population occurred between day one and day two after steaming.

From the above results, the highest bacterial abundance and diversity were found in the fresh EC samples, and some of the microorganisms may come from the “sweating” environment, the EC itself, and the operation process. The bacterial diversity and abundance of microorganisms in the Sas ware reduced after micro-steaming, which could be attributed to the high temperature environment that led to the death of some of the bacteria. The bacterial diversity and abundance of dulcimer decrease as “sweating” proceeds. These microorganisms are inhibited by specific variables and stresses, such as anaerobic environment and competition from other microorganisms, as well as fermentative metabolic environment and bacterial action (Wang et al., 2022). Over the time course of “sweating,” microbial richness and diversity showed differences at the different time points, but the differences were greatest when comparing the early and late stages. Wu et al. (2019) found a similar situation in their study of *Magnoliae Officinalis Cortex*. During the “sweating” phase, the bacterial community changed significantly.

#### 3.3.2 Taxonomic composition of microbial communities and their dynamic changes

**Figure 6:**
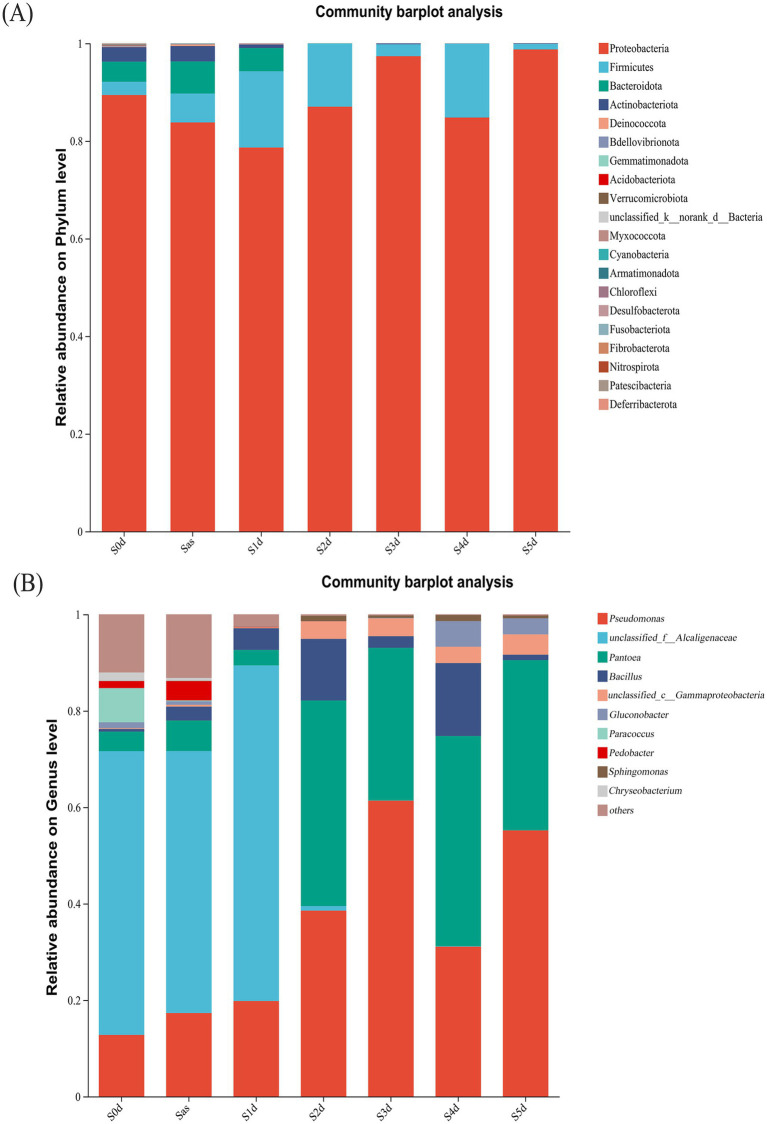
The relative abundance of bacteria at different stages of sweating phylum **(A)** and genus **(B)** in *Eucommia ulmoides*. Species with relative abundance *<*1% in the sample were classified as “others.” S0d, Sas, S1d, S2d, S3d, S4d and S5d represent the seven sampling points of the whole sweating time course, respectively.

The bacterial 16S rRNA sequence readings were sorted to obtain microbial community composition at a 97% sequence similarity level. At the phylum level, the detected sequence fragments were distributed in 20 phyla, as shown in Figure 6A. Proteobacteria, Firmicutes, Bacteroidetes and Actinobacteria were the most abundant phyla. Although some other phyla were detected, their abundance was less than 1%. Proteobacteria (84.81–98.76%) and Firmicutes (1.11–15.13%) were the dominant during the “sweating” of EC and were in constant dynamic flux. The proportions of Bacteroidetes and Actinobacteria in S0d samples were 4.13 and 2.97%, respectively. However, Bacteroidota and Actinobacteriota were almost absent on the first day after “sweating” (S1d) and were no longer be detected during the subsequent timepoints. All phyla except Proteobacteria and Firmicutes were significantly inhibited after the “sweating” treatment. Zhang et al. (2023) analyzed the composition of the endophytic microbial community in *Eucommia ulmoides* seeds and found that Proteobacteria and Firmicutes were the most abundant bacterial groups, accounting for 52.49 and 17.22%, respectively, which was similar to the results of this study. In the processing of *Eucommia* tea from *Eucommia ulmoides* leaves, dominant populations of Proteobacteria and Firmicutes in the middle and late stages were also found (Zeng et al., 2021). Meanwhile, correlation analyses indicated that bacteria may play an important role in the processing of *Eucommia* tea from *Eucommia ulmoides* leaves.

At the genus level (Figure 6B), *Pseudomonas*, *unclassified_f_Alcaligenaceae*, *Pantoea*, *Bacillus*, *unclassified_c_Gammaproteobacteria*, *Gluconobacter*, *Paracoccus*, *Pedobacter*, *Sphingomonas* and *Chryseobacterium* were the top 10 core genera (ranked in relative abundance). In S0d to S1d samples, *unclassified_f_Alcaligenaceae* (58.84%) was the absolute dominant bacteria. *Pseudomonas* (12.78%), *Paracoccus* (7.08%), *Pantoea* (4.06%), *Pedobacter* (1.46%), *Chryseobacterium* (1.74%), *Gluconobacter* (1.19%), and *Bacillus* (0.54%) and *unclassified_c_Gammaproteobacteria* (0.20%) were evenly distributed. Between S1d and S2d, there was a clear decrease in *unclassified_f_Alcaligenaceae* and a large increase in *Pantoea*. The genera Pantobacter and Pseudomonas were dominant. *Pseudomonas*, *Pantoea*, *Bacillus*, *unclassified_c_Gammaproteobacteria* and *Gluconobacter* increased gradually with the progress of “sweating,” which may be due to water leakage of *Eucommia ulmoides* during “sweating,” which is conducive to the growth and reproduction of these bacteria. The sudden disappearance of *Paracoccus* after steaming suggests that the high temperature environment brought about by steaming inhibits the presence of this microorganism. *Unclassified_f_Alcaligenaceae*, *Pedobacter*, and *Chryseobacterium* disappeared sharply on S2d of “sweating,” which may be due to the ability of some substances released from the process of “sweating” to kill these bacteria, or it may be due to cooking that causes their death. The abundance of *Pantoea* (42.65, 31.64, 43.61, 35.32%) and *Pseudomonas* (38.55, 61.35, 31.08, 55.19%) in S2d–S5d were the largest, becoming the absolute dominant bacteria genera.

Then, *unclassified_f_Alcaligenaceae* has an absolute advantage in the early stage of “sweating,” which may be related to the alkaline environment of fresh EC itself. It has been reported that common Alcaligenaceae bacteria are specialized aerobic bacteria with optimal growth temperature of 20–37°C and optimal pH of 8–10, which can produce bases from several organic acids and amides and usually do not utilise carbohydrates (Wan et al., 2014). The *unclassified_f_Alcaligenaceae* as an Alcaligenaceae bacteria belonging to Proteobacteria in the microbial taxonomy that has not yet been clearly classified to a specific genus or species, and is hypothesised to have Alcaligenaceae bacteria like functions. Then, the anaerobic environment during “sweating” is likely to be one of the main reasons for the dramatic decline of *unclassified_f_Alcaligenaceae* during the pre- “sweating” phase. A more reasonable explanation for this study is that *unclassified_f_Alcaligenaceae* is mainly present in the prediaphoretic period, probably from fresh EC, and the hypoxia environment during “sweating” causes a precipitous decline of this genus. The *Pseudomonas* genera identified in this study is not the pathogenic *Pseudomonas aeruginosa*. *Pseudomonas* has been well studied in biological control and growth promotion, and it promotes plant growth mainly through the production of plant growth hormone (Preston, 2004). In the aspect of environmental protection, *Pseudomonas* is mainly used in the degradation of chemical pesticides, wastewater treatment, and oil pollution treatment. Two strains of the genus *Pseudomonas*, BR1R-3 and BR1R-5, were isolated from inside *Brassica rapa* var. *perviridis* plants by Mari et al. (2021), and these strains enhanced inducible reactive oxygen species production in BY-2 tobacco cells. The bacteria belonging to the genus *Pantoea* have been reported to be rhizosphere and endophytic colonizing bacteria of corn and wheat plants, potato stems, rice seeds, and citrus leaves. Several studies (Tan et al., 2014; Priya et al., 2022) have reported the potential of *Pantoea* sp. as a plant growth promoter. This bacterium enhances plant growth by solubilizing phosphorus, stimulating the production of plant hormones, inducing systemic resistance, and providing protection against pathogenic microorganisms (Dastager et al., 2009). *Unclassified_f_Alcaligenaceae*, *Pseudomonas*, and *Pantoea* were abundant during “sweating” and presumably have a great influence on the entire “sweating” process.

### 3.4 Correlation analysis between microbial community, metabolites and main components

**Figure 7:**
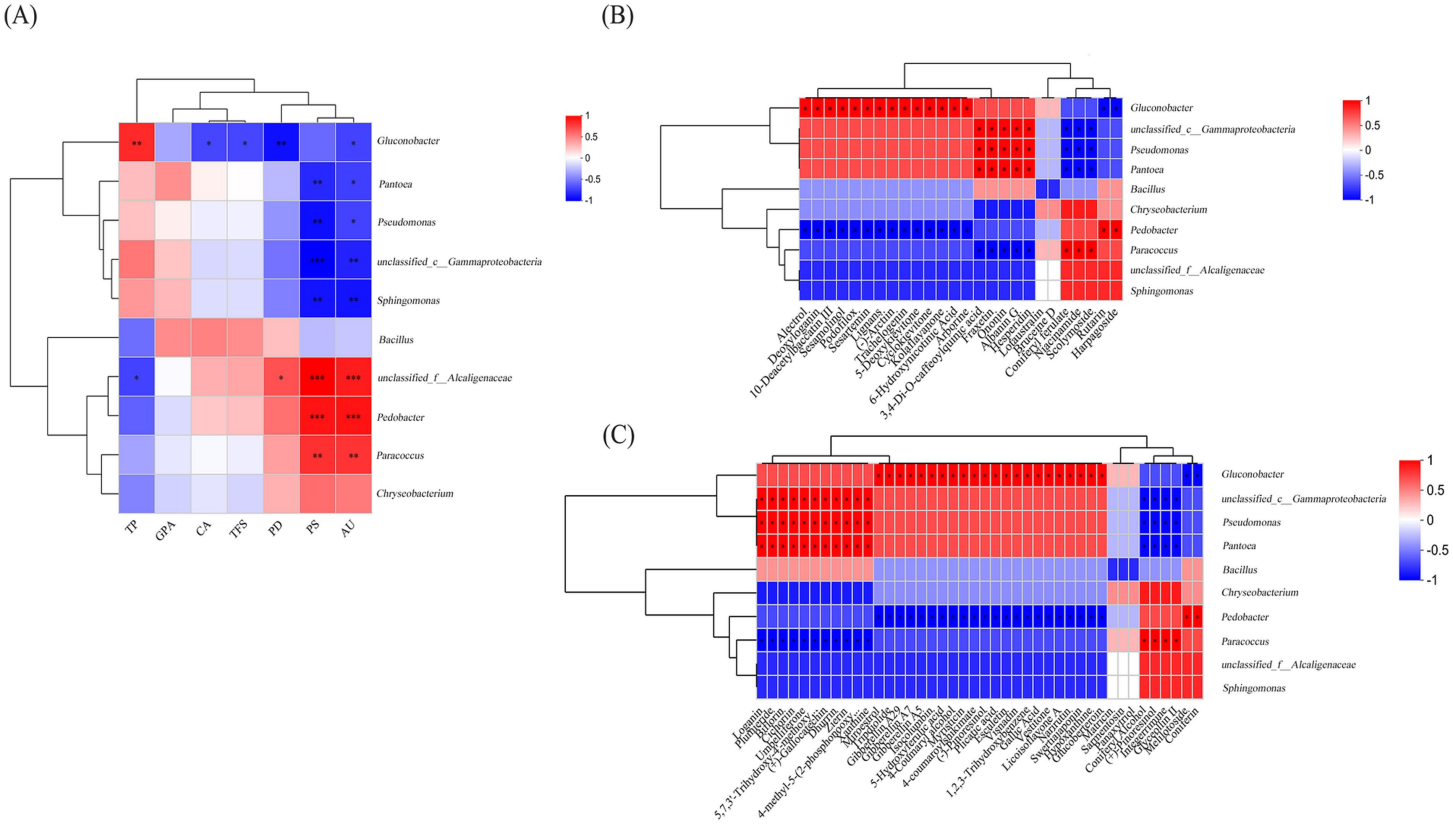
Heat maps of the relative abundances of the top 10 bacteria genera level communities and correlations between the seven active ingredients **(A)**, differential metabolites in the positive-ion mode **(B)** and the differential metabolites of negative ions **(C)**, significant correlation coefficients are noted in * where *p* < 0.05. *, ** and *** denote significant differences at *p* < 0.05, *p* < 0.01, and *p* < 0.001, respectively.

In order to explain the role of “sweating” in the quality formation of EC, this study analyzed the potential correlation between bacteria, metabolites, and major chemical constituents in samples of EC samples. The results showed that the main chemical constituents formed strong and extensive co-occurrence relationships with secondary metabolites and bacteria (Figure 7). The change of active ingredients during the “sweating” process is related to microbial metabolism (Cruxen et al., 2019). Figure 7A illustrates a heat map of the 10 core bacterial genera and seven active ingredients and correlation clustering. The contents of AU and PS were significantly positively correlated with the three bacteria (*unclassified_f_Alcaligenaceae*, *Pedobacter*, and *Paracoccus*). It was negatively correlated with *unclassified_o Enterobacterales*, *Pantoea, Pseudomonas* and *unclassified_c_Gammaproteobacteria*. Bacterial *Gluconobacter* was significantly negatively correlated with AU, PD, TFS, and CA, while demonstrating a significant positive correlation with TP. *Unclassified_f_Alcaligenaceae* is significantly positively correlated with PD, and negatively correlated with TP. This preliminary data suggests that the microbiota significantly associated with the active ingredient is associated with changes in the active ingredient during “sweating.” However, the role of these microbiota in EC “sweating” needs to be further analyzed in monoculture fermentation studies.

Furthermore, the specific role of microorganisms in the “sweating” process of EC merits further investigation. Figures 7B,C show that secondary metabolites can be affected by the structure of the microbial community. Figure 7B shows the heat map of the correlation between the differential metabolites and the relative abundance of the top 10 bacterial genera in the positive ion mode. Twelve differential secondary metabolites, including alterol, deoxyglycine, 10 deacetylbacartin III, sesteramine, lignan, (−)-arctiin, airway poietin, 5-deoxyvidone, cyclovidone, corlafarone, 6-hydroxynicotinic acid, and arbiflorin, were found to be positively correlated with *Gluconobacter*. However, it was negatively correlated with *Pedobacter*, and the above substances were significantly up-regulated in the “sweating” process of EC, indicating that *Glucobacterium* is beneficial bacteria in the “sweating” process of EC. 3,4-biso-caffeoylquinic acid, esculin, ononin, albanin G and hesperidin were positively correlated with *unclassified_c_Gammaproteobacteria*, *Pseudomonas* and *Pantoea*. While negatively correlated with *Parecoccus*, these secondary products were also up-regulated substances during the process of “sweating.” On the contrary, coniferyl ferulate, nicotinamide and scopolamine were positively correlated with *Parecoccus*, but negatively correlated with *unclassified_c_ Gammaproteobacteria*, *Pseudomonas* and *Pantoea*. Rutalin and Habargoside were positively correlated with *Pedobacter*, but negatively correlated with *Gluconobacter*. Figure 7C shows the heat map of the correlation between the differential metabolites and the relative abundance of the top 10 bacterial genera in the negative ion mode. The results showed that 11 differential metabolites, including brucine, plumid, dichlorobenzene, chicoritin, umbellone, durin, Zilin, xanthine, (+)-gallic acid, 5,7,3′-trihydroxy-4′-methoxyflavanone, and 4-methyl-5-(2-phosphooxyethyl) thiazole were significantly correlated with the serum levels of the two metabolites. Specifically, *unclassified_c_Gammaprotebacteria*, *Pseudomonas* and *Pantoea* were positively correlated, while *Paracoccus* was negatively correlated. Additionally, miloestrol, triptolide, gibberellin A29, gibberellin A7, gibberellin A5, isogulenbine, 5-hydroxyferulic acid, 4-coumaryl alcohol, myristin, 4-coumaryl potassium bisulfate, (−)-pinoresinol, folding acid, aesculetin, visnatin, 1,2, 3-hydroxybenzene, gallic acid, glycyrrhizin, licorice flavone A, naringin, swertioside, hypoxanthine and glucoside were positively correlated with *Gluconobacter*, but negatively correlated with *Pedobacter*. Coniferinol, (+)-piniferinol, integrin imine and glycyrrhizin II were positively correlated with *Paracoccus* and negatively correlated with *unclassified_c_Gammaproteobacteria*, *Pseudomonas* and *Pantoea*. Luteolin and coniferinin were positively correlated with *Pedobacter* and negatively correlated with *Gluconobacter*. *Thus, Gluconobacter, Pedobacter, unclassified_c_Gamma-proteobacteria, Pseudomonas, Pantoea* and *Parecoccus* were the dominant genera in the process of “sweating.” The above analysis showed that *Gluconobacter*, *unclassified_c_Gammaproteobacteria*, *Pseudomonas*, and *Pantoea* were positively correlated with significant up-regulation and negatively correlated with significant down-regulation during “sweating.” These four genera are the beneficial genera of EC during “sweating.”

To further investigate the metabolic relationships of differential components before and after “sweating” in EC, the metabolic pathways of secondary metabolites were analyzed. Table 4 shows that ononin, esculin, gallic acid, coniferyl aldehyde, umbelliferone, coniferyl alcohol, and 4-coumarinol are mainly involved in the biosynthetic pathways of other metabolites, including isoflavone biosynthesis, flavonoid biosynthesis, and phenylpropanoid biosynthesis. 6-Hydroxynicotinic acid, nicotinamide and 4-methyl-5-(2-phosphooxyethyl) thiazole are involved in the metabolism of cofactors and vitamins, mainly niacin and nicotinamide metabolism and thiamine metabolism. lotoastaline and durin are involved in the metabolism of cyanoamino acids. Deoxyglycine, 10 deacetylbacartin III, brucine and gibberellin are involved in the metabolism of terpenoids and polyketones, including monopeptide biosynthesis and diterpenoid biosynthesis. Xanthine and hypoxanthine are involved in nucleotide metabolism. It can be speculated that *Gluconbacter*, *unclassified_ c_Gammaprobacteria*, and *Pseudomonas* mainly affect the metabolic pathways of nicotinic acid and nicotinamide metabolic pathways, cyanogenic amino acid metabolism, flavonoid biosynthesis, isoflavone biosynthesis, phenylpropane biosynthesis, monoterpene biosynthesis, and diterpene biosynthesis. These effects lead to the changes in alkaloids, amino acid-related compounds, flavonoids, phenylpropanoids, and terpenoids during the process of “sweating” in EC. The primary process of “sweating” is similar to fermentation, in which the samples are exposed to a certain amount of heat and moisture, and the quality of the samples is improved after “sweating.” Bacteria with significant changes in relative abundance were associated with changes in active ingredient content and metabolite content caused by the “sweating” treatment of EC. Based on the analysis of the microbial community during the “sweating” process of *Angelicae Sinensis Radix*, the relative abundance of *Enterobacter*, *Klebsiella*, *Clostridium welchii* and *Candida* favoured the quality of *Angelicae Sinensis Radix* (Wu et al., 2019). The formation of the “strong odour and brownish inner surface” characteristic of *Magnoliae officinalis cortex* after “sweating” may be related to the metabolism of microbial communities such as *Aspergillus* and *Pseudomallei Candida* (Wei et al., 2019). “Sweating” improves the quality of the herbal medicine EC. The present study suggests that “sweating” creates favourable conditions for the reproduction of functioning bacterial genera, which leads to an increase in their abundance.

**Table 4:** Metabolic pathways of differential components.

Metabolite	Secondary metabolic pathways	Description of pathways
Ononin	Biosynthesis of other secondary metabolites	Isoflavone biosynthesis
Podofilox	Biosynthesis of other secondary metabolites	—
Scolymoside	Biosynthesis of other secondary metabolites	Flavonoid and flavonol biosynthesis
Fraxetin	Biosynthesis of other secondary metabolites	—
Gallic acid	Biosynthesis of other secondary metabolites	—
(+)-Gallocatechin	Biosynthesis of other secondary metabolites	Flavonoid biosynthesis
Coniferyl aldehyde	Biosynthesis of other secondary metabolites	Phenylpropane biosynthesis
Vestitone	Biosynthesis of other secondary metabolites	Isoflavone biosynthesis
Glyceollin II	Biosynthesis of other secondary metabolites	Isoflavone biosynthesis
4-Coumaroylshikimate	Biosynthesis of other secondary metabolites	Flavonoid biosynthesis
Glucoberteroin	Biosynthesis of other secondary metabolites	2-oxo-carbonyl acid metabolism
Esculetin	Biosynthesis of other secondary metabolites	—
Umbelliferone	Biosynthesis of other secondary metabolites	—
Melilotoside	Biosynthesis of other secondary metabolites	—
Coniferyl alcohol	Biosynthesis of other secondary metabolites	Phenylpropane biosynthesis
5,7,3′-Trihydroxy-4′-methoxyflavanone	Biosynthesis of other secondary metabolites	Flavonoid biosynthesis
(+)-Pinoresinol	Biosynthesis of other secondary metabolites	—
Xanthine	Biosynthesis of other secondary metabolites	Purine metabolism
4-Coumaryl alcohol	Biosynthesis of other secondary metabolites	Phenylpropane biosynthesis
5-Hydroxyferulic acid	Biosynthesis of other secondary metabolites	Phenylpropane biosynthesis
Coniferin	Biosynthesis of other secondary metabolites	Phenylpropane biosynthesis
6-Hydroxynicotinic acid	Metabolism of cofactors and vitamins	Niacin and nicotinamide metabolism
Niacinamide	Metabolism of cofactors and vitamins	Niacin and nicotinamide metabolism
4-methyl-5-(2-phosphonooxyethyl)thiazole	Metabolism of cofactors and vitamins	Metabolism of thiamine
Lotaustralin	Metabolism of other amino acids	Cyanoamino acid metabolism
Dhurrin	Metabolism of other amino acids	Cyanoamino acid metabolism
10-Deacetylbaccatin III	Metabolism of terpenoids and polyketones	Diterpenoid biosynthesis
Deoxyloganin	Metabolism of terpenoids and polyketones	Monoterpene biosynthesis
Gibberellin A5	Metabolism of terpenoids and polyketones	Diterpenoid biosynthesis
Gibberellin A7	Metabolism of terpenoids and polyketones	Diterpenoid biosynthesis
Gibberellin A29	Metabolism of terpenoids and polyketones	Diterpenoid biosynthesis
Loganin	Metabolism of terpenoids and polyketones	Monoterpene biosynthesis
Xanthine	Nucleotide metabolism	Purine metabolism
Hypoxanthine	Nucleotide metabolism	Purine metabolism

## 4 Conclusion

This study demonstrated that the structure of the bacterial community plays a crucial role in the chemical transformation and quality formation during the “sweating” process of EC. High-throughput sequencing and chemical analysis revealed significant correlations between bacterial communities, primary chemical components, and secondary metabolites. The key genera, *Gluconobacter, Pedobacter, Pseudomonas, Pantoea and Parecoccus* are identified as critical drivers in this process. These findings highlight microbial regulation as a fundamental factor in EC quality development. Further studies should validate these results through systematic research on the microbial community changes in EC of different origins. Meanwhile, the enzymatic mechanisms to clarify the microbial contributions to bioactive compound formation during EC processing should be further explored in subsequent studies.

## Data Availability

The datasets presented in this study can be found in online repositories. The names of the repository/repositories and accession number(s) can be found at: https://www.ncbi.nlm.nih.gov/, PRJNA1216076.
